# PVA/chitosan hydrogels loaded with octenidine and 2-phenoxyethanol for wound dressings

**DOI:** 10.1080/07853890.2021.1896910

**Published:** 2021-09-28

**Authors:** Edoardo Massarelli, Ana Paula Serro, Benilde Saramago

**Affiliations:** a CMIC, Politecnico di Milano, Milano, Italia; b CQE, Instituto Superior Técnico, Universidade de Lisboa, Lisboa, Portugal; c Centro de Investigação Interdisciplinar Egas Moniz (CiiEM), Egas Moniz Cooperativa de Ensino Superior, Caparica, Portugal

## Abstract

**Introduction:**

Polyvinylacohol (PVA)/chitosan hydrogels are ideal candidates for the production of wound dressings as their combination can provide good mechanical and antibacterial properties, alongside the ability to be loaded and release drugs [1,2]. The main goal of this work is to evaluate the possibility of using PVA/chitosan hydrogels as platforms for the release of octenidine dihydrochlorate and 2-phenoxyethanol, a drug combination that has antiseptic, antibacterial and antimycotic properties.

**Materials and methods:**

PVA aqueous solutions were prepared by dissolution at 90 °C and chitosan was added to get different mass ratios (3:1, 1:1) and a final polymer concentration of 5% w/w. The mixtures were poured in petri dishes and submitted to five cycles of freeze-thawing (18 h at −20 °C, 6 h at 23 °C in each cycle) to trigger polymerisation. After a washing step, they were lyophilised. Before testing, the samples were hydrated for 72 h. Swelling ratio, water content and degradation experiments in simulated exudate containing lysozyme were performed to assess the stability of the hydrogels. Contact angle was measured using captive bubble method and hydrogels structure was assessed using SEM. Drug loading was carried out by soaking the samples in Octiset® solution at room temperature. Drug release experiments were performed in simulated exudate using Franz cells.

**Results:**

The hydrogels showed swelling ratios between ≈1300 and 2100% and high water contents of 93–95%. Degradation in the first 2 days in the presence of the protein was less than 37%. The hydrogels showed high hydrophilicity and porosity. Drug loading and release experiments (Figure 1) proved that the hydrogels are able to release both drugs in a controlled way during the first day. **Discussion and conclusions:** The obtained results suggest that the proposed formulations possess drug retention abilities compatible with their use. They shall be suitable for the production of wound dressings with therapeutic properties. Further research should provide insight into the mechanical properties of the hydrogels, their antibacterial behaviour and the choice of a sterilisation method.

**Figure 1.**
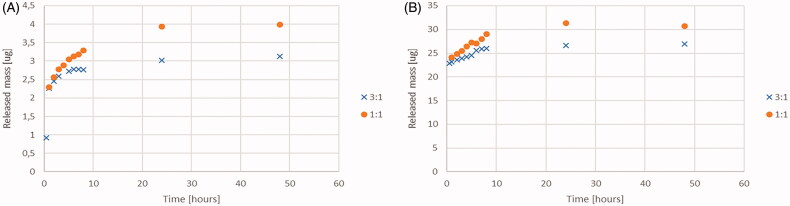
Cumulative drug release curves of octenidine dihydrochlorate (A) and 2-phenoxyethanol (B).
